# Lectures on the Exploration and Treatment of Diseases of the Chest

**Published:** 1840-05-16

**Authors:** 


### MEDICAL EXAMINER.

DEVOTED TO MEDICINE, SURGERY, AND THE COLLATERAL SCIENCES.

No. 20.] PHILADELPHIA, SATURDAY, MAY 16, 1840. [Vol. III.

### LECTURES ON THE EXPLORATION AND TREATMENT OF DISEASES OF THE CHEST.

Lecture I.—*Uses of Classification in the Study of Disease—Comparison of Physical and General Signs.* By W. W. Gerhard, M. D.

In the study of diseases of the chest, as well as those of other cavities of the body, the classification adopted for the convenience of the pupil must often be arbitrary and imperfect. Diseases which are naturally closely connected together are separated into artificial subdivisions, while others which are essentially dissimilar are brought together in a manner which tends to lead the mind of the pupil from the study of their true relations; still, the artificial divisions which have been admitted for a long time, are in general the most easily recollected, and for purposes of study possess many advantages. While the erroneous conclusions to which an artificial classification sometimes leads may be obviated by attention to real points of similarity, and the natural connexion may finally be re-established after it has been for a time broken up. There is thus a double task for the author or teacher: first, that of analysis, for the purpose of discovering and pointing out isolated facts; next, that of synthesis, or of bringing together those which present, in common, strongly marked fundamental characters. Both of these tasks must be kept in view, or the true scientific connexions of disease may be forgotten, and the diagnosis will be merely local, and therefore imperfect, instead of being based on correct and enlarged data.

The very division of diseases into those belonging to the several cavities of the body, is, in a great degree, artificial; and although many of these affections are strictly local, or nearly so, there are others which are nothing more than evident shoots from a diseased root, which extends itself widely throughout the body. In these cases the disease, when it shows itself in the chest, is readily recognised, and is taken as a sign of the general disorder. Sometimes the local disorder grows rapidly, and becomes the prominent malady; but in all such cases it is only a sign of a deeper and more extended mischief.

The remarks which are applicable to the study of disease, are naturally extended to the means of discovering it. These are separated according to the various methods of investigation resorted to, whether founded on the general symptoms, or the physical changes of the part; and they are more or less deceptive without a careful comparison, or collation of them, one with another. This is not done by a smatterer in pathology or in diagnosis; hence the conclusions attained by him are uncertain, and cast discredit upon medicine, and especially on exact diagnosis. The rules of true philosophy, as applied to medicine, do not differ from those of any other science. The same mental discipline, and the same rules of philosophizing are required; and, with equally ascertained data, the same degree of certainty may be acquired. We must first separate a group of facts into detached elements, examine them in every practicable way, and then bring them together again, and reinstate them in a regular and natural order.

In all departments of science, especially the natural sciences, with which medicine is so closely connected, we examine the objects of our research in two ways: first, as actually existing; and, secondly, as past. The objects actually present, are known by evident signs; those past, are discoverable by the more obscure traces which they have left behind. We thus discern the modes of a disease, just as we learn the habits of a plant or an animal, from its footsteps, or the remains of its food; and we can, in either case, learn the peculiarities which have characterized them during life, or during the continuance of the diseased action, by the phenomena actually occurring, or the changes consequent upon them. We are therefore obliged to discriminate in the study of disease, between those phenomena which are actually going on, and those which have terminated and are no longer of mischief; this obviates many grave errors, and prevents a disorder from being confounded with the lesions which often constitute the proof of its cessation, or it prevents us from mistaking the actions which are in their nature mischievous for those which are useful and salutary. The latter error is one of more frequent occurrence than the former, for the severe diseased action may require for its removal a slighter, but more permanent deviation from the healthy functions of the part. And although this secondary disorder would, if occurring singly, constitute a disease, it ceases to be one when it is merely a curative agent which exerts a favourable influence with the primary affection. These diseases are often very similar to blisters, or other powerful revulsive means, truly curative, and they then only become injurious when heedlessly meddled with.

The recognition both of the primary and secondary disorders, is more easy in affections of the thorax than in those of other cavities of the body, from our possessing the advantage of two distinct modes of investigation. These are the altered functions, including both those of the viscera of the thorax and of the rest of the body, and the physical signs offered by the diseased organ. The general symptoms of thoracic diseases are learned in the same way as those of other organs of the body; but the physical signs, on the other hand, are so much more applicable to chest affections than to any other disorders, that they are, in practice, with a few exceptions, used only in the diagnosis of the diseases of this cavity, and are generally described with direct reference to the chest. This peculiar fitness of the physical signs for the study of pectoral diseases, depends upon the conformation of the thorax and the structure of the organs contained within it. These are important viscera,—the lungs and heart, which are organs possessing different degrees of density, and constantly in motion; hence we may, from the examination of the thorax, not only ascertain the density of the organs when at rest, but we may, with great certainty, discover whether they act in a regular and natural manner, and what impediments interfere with their motion. These advantages are not offered by the viscera of any other cavity; for although the physical properties of them are sometimes sufficiently marked to enable us to detect variations in form, or in density of tissue, they are never susceptible of sufficient motion to cause an audible sound by their own contraction, or by the passage of a fluid throughout their cavity. The physical signs are, therefore, chiefly adapted to the investigation of the diseases of the thorax.

Physical exploration is much more extended in its application when combined and compared with the rational signs, than if used alone. For in itself it teaches us rather the condition of organs as modified by disease, than the manner in which the disease forms, or the mode in which it advances. This is especially the case in the chronic diseases of the chest, which depend upon a general vice of the economy, for in order to distinguish disease from health, by the physical exploration of the lungs and heart, it is necessary that a change should occur in the structure of the tissue,—and as this alteration is only brought about slowly and gradually, we cannot always decide whether the tissue is or is not diseased to some extent, if it be in a degree not sufficient to produce an important change in the conformation of the part. There is, therefore, no means of arriving to a correct conclusion in the diagnosis of pectoral diseases, other than a union of the two modes of investigation, which will then work together as two different ways of arriving at the same end. When physical exploration is properly understood, and compared with the symptoms, it will be found to be even more useful for its negative than its positive results, that is, it will be more useful as a means of showing that some diseases do not exist, or that a given disease is not arrived at a point of structural disorganization sufficient to endanger life, than as direct evidence of the mischief already done to the organs. The positive evidence derived from physical exploration is so simple and easily discovered, that after acquiring a certain familiarity with it, little attention is required to discover the full value of the signs; the negative evidence, on the other hand, is much more difficult, for a thorough knowledge of the means of examination, and much practice in using them, are required to pronounce with certainty as to the existence of slight alterations of a part, or the absence of decided structural change. But when the necessary familiarity is acquired, the certainty of the knowledge obtained from this source is such, that we may rely upon its negative evidence as confidently as upon its positive signs, especially when compared with the indications derived from the general symptoms.

The great value of the negative evidence of physical exploration depends upon its certainty. The process of reasoning which renders negative evidence of value in diagnosis, is called reasoning by way of exclusion; but, although it is of great utility when skilfully applied, it is useless unless a disease is announced by positive signs when it exists, and then we may look upon the absence of these signs as a proof that it is not present. If, on the other hand, the signs themselves be doubtful, the absence of them is of course no proof that the disease does not exist; or if these signs be of such nature that we can ascertain them with extreme difficulty, they lose the advantages of serving both as negative and positive evidence. Now, in the diseases to which physical exploration is applicable, this is not the case. The signs are, in general, very easily ascertained, and are always, under similar circumstances, the same; hence they may be used in the way of exclusion with great confidence; that is, when they are not discovered by one who is familiar with the means of exploring them, they may be confidently said not to exist. This negative evidence, as I have already stated, is useful in two ways: first, as evidence that there is no disease; secondly, as evidence that there is no great change of structure. The first requires that the general symptoms should agree, as it were, with the physical signs, in proving the integrity, or the comparative soundness of the part. The second requires that the physical signs should be, to a certain extent, contradicted or disproven by the general symptoms. But, as this seeming discrepancy is applicable only to the degree of the alteration, and not to its nature, there is, in fact, no real contradiction between the two means of examination. On the contrary, they will be found, when compared together, to accord singularly in the principal deductions which are drawn from each of them.

The extent of application of the physical means of exploration is, perhaps, novel to many persons who are not familiar with the beautiful application of the laws of diagnosis, by way of exclusion, in which the certainty of the physical signs renders them even more useful, than in other cases in which a precision which approaches mathematical correctness is required; but this very certainty may render them an occasional source of error with those who are neither accustomed to their use, nor perfectly familiar with the ordinary symptoms of disease. That is, an art which is evidently based upon fixed physical laws, may lead to error when the data upon which the problem of diagnosis is founded are not correctly settled, although the process of reasoning may still be the correct one. But, the abuse of a certain method of observation does not constitute a real objection to its employment; it merely proves that it is necessary to surmount the first difficulties which attend its acquisition.

The great importance of the comparison of the general symptoms and physical signs has become more apparent with the more general employment of physical exploration, as a really practical aid to diagnosis. The earlier writers on auscultation, especially Laennec, were rather disposed to separate physical from symptomatic diagnosis: this error depended upon the novelty of the art, and the overstrained efforts to extend its application,—but as physicians became more familiar with it, and had opportunities for testing its merits, it was placed on its real footing, and regarded as the most useful of all symptoms taken singly, but as neither the only class of symptoms to be relied upon, nor as superseding the general signs. We are indebted to the French pathologists for pointing out the necessity of comparison of all the symptoms of pulmonary disease, and of connecting this comparison with their succession or order. It is a subject largely insisted upon in the writings of Andral, but one which was most completely developed in the lectures of Dr. Louis. It afterwards received much attention from Dr. Stokes, and others, who have occupied themselves with the study of pectoral diseases.

Although it is not at this time necessary to insist upon the truth of the physical signs to those who are conversant with them, their certainty may still appear questionable to a few who are not practically acquainted with them. As these signs are based upon the settled laws of physical science, and in fact involve some universally admitted principles, the only reason for doubting their accuracy is a want of due knowledge on the subject. But as a certain acquaintance with them is necessary to appreciate the evidence upon which they depend, I may properly enough point out what is included under the terms physical exploration and physical signs. Physical exploration includes the modes of ascertaining the changes which occur in the physical structure of organs; these changes we appreciate by the alterations of form, and by the sounds produced in the interior of the body by the motion of solids, or of elastic or non-elastic fluids, or by the resonance which is yielded by the surface when tapped or struck by the finger. This latter mode of examination obviously depends upon the different density of organs, and in the cavity of the thorax, chiefly upon the existence of air, and the percussion is more or less clear or dull, as the quantity of air contained within the thorax is greater or less. The alterations of form are few in number, and are readily learned; but the signs dependent upon changes in the sounds produced by the passage of the air and of the blood within the thorax, or the resonance of the air when thrown into motion by the act of speaking, seem comparatively difficult, and involve more complicated phenomena. The same remarks are applicable to the signs of percussion, which are not a little difficult; but in either case, the sounds are regularly and uniformly the same under similar circumstances. The difficulty in learning the physical signs consists in two distinct points; first, in the acquisition of the sounds themselves, considered as simple phenomena; secondly, in the knowledge of the condition of the organs which corresponds to these sounds. The sounds themselves require not only to be learned well enough to be understood, but they must be fixed so thoroughly in the mind that no room should be left for mistaking one for another. This demands time, attention, and organs of hearing which are not physically incapable of discriminating between sounds which at first may seem nearly similar. The first difficulty is surmounted; there still remains the other, which requires a knowledge of many circumstances which are connected with the pathology of the disease. These are those which relate to the physical condition of the organs or the pathological anatomy of the parts, and to the functional action of the organs which is necessary for the production of most of the sounds. Hence the knowledge of the physical means of exploration requires no little time and attention, and cannot be learned in a careless or hasty manner.

There is, therefore, this impediment to the study of the diseases of the chest, and of physical exploration, that the act itself is a matter of difficulty, and requires more labour than is willingly bestowed upon it. The whole process of investigation requires this attention; even the manual or mechanical precautions necessary to be taken are not to be learned at the first trial, but require time in the performance of this art, like that of every other; for the ear and hand are not at first capable of the delicate and varied actions necessary for the satisfactory exploration of the thorax. But, although the apparently complex nature of physical exploration may prevent many from attempting its study, the difficulties which at first present themselves are readily enough removed by patient and laborious attention, and are more than compensated by the certainty which results from a mode of investigation based upon fixed physical laws. Every step in the acquisition of this knowledge is appreciable, and in proportion as it becomes more accurate, the diagnosis of disease assumes a new character, which is never acquired when confined to the functional symptoms. This is true even at present, when the comparison of local and general symptoms to which I have just alluded has rendered the latter much more clear, and their value better defined. The physical signs have served as a point of departure, with which to compare the rational symptoms, and have thus rendered the latter more easy of recognition, and more positive in their relations with the internal lesions of the thorax. This is so obviously the case, that a glance at the works of any of the later writers upon the subject is sufficient to show that the rational signs have become of more practical service for the study of diagnosis than they have ever been before, and that many of these symptoms have been investigated with a care which was never before bestowed upon them. Some symptoms, it is true, have fallen into comparative neglect, because they are no longer of decided utility in diagnosis, but the greater number have derived new value from their connexion with the physical signs.

As the diagnostic characters of the diseases of the chest are composed of several distinct sets of symptoms, they may be studied after each class of symptoms has been separately learned, or the diseases themselves may be first observed, and the symptoms analyzed as they present themselves at the bedside. The former method is naturally adopted in a systematic treatise, or course of lectures; the latter belongs more properly to clinical or demonstrative medicine,—a subject of which I treat more at large in another place. As the object of the present series of lectures is not only to explain the mode of application of these methods of investigation to the study of disease, but to teach the methods themselves, it resolves itself naturally into two parts. The first part will contain the explanation of the physical signs, and teach the method of acquiring them which I have found most convenient for the pupil. In connection with this portion of the lectures, I shall treat of those functional symptoms which are immediately connected with the organs of the thorax, and are therefore most conveniently studied at an early part of the course. The second part will be devoted to the study of individual diseases in connection with their symptoms and treatment. The series will thus comprise, as nearly as my time will permit, a complete history of the modes of exploration used in the diseases of the thorax, and of those diseases themselves.

The difficulties which attend the study of pectoral diseases, depend more, however, upon an imperfect method, than upon the subject itself, and may be obviated in a great degree by adopting an order which is in harmony with the natural connexion of these signs. In all essential particulars they are readily understood when they are pointed out by one who is practically familiar with them; one who is yet unpractised becomes embarrassed when he examines a patient without the aid of an adviser. Signs which are really different are sometimes confounded together, and those which are mere varieties of the same species are thought to be perfectly distinct. If the signs are well characterized sounds, their discrimination should always be easy, and error would be impossible. That is, the correspondence between the sounds in certain physical conditions is necessarily exact, and the chances of error depend upon an erroneous interpretation of them. The interpretation is very different from the recognition of the sounds, and necessarily includes more data and more complicated reasoning; but there can be no reason for not detecting a sound connected with the chest; it should be at least as easily recognised as the tone of voice or spoken language; it does not require any peculiar nicety of organs, or a finely cultivated musical ear, but merely a good organ, and the attention necessary for observation of any natural phenomena. I shall endeavour to arrange the physical signs in such an order as will facilitate this part of your study, and shall explain the method of acquiring them which you will find most convenient. The great secret is to give great attention to the signs at first, and fix each one in your mind as you go on. If you content yourselves with detecting them when pointed out to you, and merely understanding the differential characters without actually knowing them, you will gain but little, and you will never acquire the knowledge of them which is practically useful. The best method of avoiding the habit of careless observation, is to dwell long upon each sign at first, and afterwards connect it with others which are closely related to it, and are met with either in the same or in other patients. The whole matter will in this way be rendered singularly easy.

